# PTEN-deficient, chromosomal instability colorectal cancer is hypersensitive to STAT3 inhibition

**DOI:** 10.7150/ijbs.111254

**Published:** 2025-10-20

**Authors:** Guowen Ren, Yue Pu, Xiumei Zhang, Jinghong Chen, Eun Ju Yang, Shishi Tao, Li-Jie Chen, Wenli Zhu, Kin long Chan, Guanghui Luo, Chuxia Deng, Joong Sup Shim

**Affiliations:** 1 Cancer Centre, Faculty of Health Sciences, University of Macau, Taipa, Macau SAR, China.; 2 Institute of Cancer Research, Shenzhen Bay Laboratory, Shenzhen 518132, China.; 3 Laboratory Animal Center, Guangzhou Medical University, Guangzhou, China.; 4 Kiang Wu Hospital, Macau SAR, China.; 5 MOE Frontiers Science Center for Precision Oncology, University of Macau, Taipa, Macau SAR, China.

**Keywords:** Colorectal cancer, PTEN, STAT3, PLK1, Synthetic lethality, chromosomal instability

## Abstract

Genetic alterations that induce chromosomal instability (CIN) in colorectal cancer (CRC) cells result in partial impairments in a crucial cellular process, which present an opportunity for therapeutic exploitation in cancer treatment. In our effort to identify therapeutic vulnerability in PTEN-deficient CRC, we found that PTEN-deficient CRC cells exhibited elevated CIN phenotype and were hypersensitive to STAT3 inhibition. STAT3 inhibition induced a high level of abnormal spindle formation, causing mitotic arrest and death in PTEN-deficient CRC cells. Mechanistically, PTEN deficiency led to an increased phosphorylation in STAT3 and the hyperactivation of the downstream mitotic kinase PLK1, resulting in the formation of abnormal mitotic spindles and CIN. Inhibition of STAT3 strongly suppressed PLK1 phosphorylation in a STMN1-dependent manner, further inducing mitotic abnormalities in the cells. This irreparable mitotic defect triggered hyperactivation of the spindle assembly checkpoint and mitotic cell death in PTEN-deficient CRC cells. Collectively, our findings suggest that targeting STAT3-PLK1 axis represents a novel therapeutic approach for CRC cells with PTEN loss.

## Introduction

Colorectal cancer (CRC) is one of the most common cancers worldwide, arising from normal epithelium through a series of genetic events. Within these genetic events of CRC, genomic instability is considered as an essential feature, which is characterized by chromosomal instability (CIN), CPG island methylator phenotype (CIMP) and microsatellite instability (MSI) 1. CIN is a characteristic feature evident in a substantial proportion, ranging from 65% to 70%, of sporadic CRCs 2. CIN results in losses and gains of entire chromosomes or large regions within a chromosome 3. Alterations in CIN causing genes lead to chromosomal aneuploidy, accompanied by the sub-chromosomal amplifications and a pronounced incidence of loss of heterozygosity (LOH), which enable cells to accumulate genetic changes facilitating cancerous progression. The presence of CIN or alterations in CIN causing genes in CRC may render cancer cells higher susceptibility to certain targets, such as metabolic stress 4, thereby presenting an opportunity for therapeutic exploitation.

Phosphatase and tensin homolog (PTEN) are one of the tops frequently mutated tumor suppressors in various types of cancer. PTEN controls several critical cellular processes, such as cell cycle progression, proliferation and survival, through regulating various downstream targets. PTEN is primarily known for its lipid phosphatase activity, which dephosphorylates phosphatidylinositol-3,4,5-trisphosphate (PIP3) and consequently suppresses the PI3K/AKT pathway. However, emerging evidence underscores PTEN's essential role in maintaining chromosomal stability and integrity, which is independent of its role in the PI3K/AKT pathway. PTEN physically interacts with centromeric proteins, such as centromere-specific binding proteins (CENP-C) to protect centromere stability 5. Furthermore, PTEN directly dephosphorylates and inhibits mitotic kinase polo-like kinase 1 (PLK1) to control polyploidy 6. In addition, PTEN is recruited to centrosomes and mitotic spindles by PLK1 to create docking sites for DLG1 and EG5, promoting symmetrical chromosomal segregation 7, 8. Loss of PTEN compromises mitotic spindle architecture and spindle pole motility, leading to chromosome misalignment, mis-segregation, mitotic catastrophe, and tumorigenesis. Notably, the dysregulation of PTEN function is frequently observed in CRC, which facilitates CIN during colorectal neoplasia 9-11. Therefore, the CIN phenotypes resulting from PTEN deficiency in CRC would provide a therapeutic opportunity for CRC treatment.

In our effort to discover therapeutic vulnerability in CRC cells with PTEN loss, we previously conducted synthetic lethal drug screen for PTEN-deficient CRC and identified STAT3 inhibitor as one of the top candidate synthetic lethal drugs 12, 13. In the present study, we comprehensively analyzed the synthetic lethal interaction between PTEN and STAT3 with in-depth mechanistic study in CRC cells. We herein report that PTEN-deficient CRC cells have activated STAT3 signal, followed by PLK1 hyperactivation, leading to abnormal mitotic and CIN phenotypes in the cells. Inhibition of STAT3 strongly suppressed PLK1 phosphorylation in PTEN-deficient CRC cells, inducing severe mitotic abnormality, leading to the spindle assembly checkpoint (SAC)-mediated mitotic death. Taken together, this study provides STAT3-PLK1 axis as a potential therapeutic vulnerability in PTEN-deficient CRC cells with chromosomal instability.

## Materials and Methods

### Cell culture and reagents

The human colorectal cancer cell lines HCT116, DLD1 and RKO were obtained from ATCC (American Type Culture Collection). HCT116 cells were cultured and maintained in RPMI 1640 medium (Thermo Fisher Scientific) supplemented with 10% fetal bovine serum (Thermo Fisher Scientific, Waltham, MA), in a humid environment of 5% CO_2_ at 37 °C. RKO cells were cultured in Dulbecco's Modified Eagle's medium (DMEM) (Thermo Fisher Scientific) supplemented with 10% FBS at 37 °C with 5% CO_2_. All the cell lines were routinely tested and confirmed negative for mycoplasma by the iPSC Core facility in the Faculty of Health Sciences, University of Macau (https://fhs.umac.mo/research/ipsc-core/). Stattic (DC7613) and Volasertib (DC7187) were purchased from DC Chemicals (Shanghai, China).

### Cell viability assay and proliferation assay

Cells were seeded in 96-well plates at 3,000 cells per well or in 24-well plates at 250,000 cells per well with either vehicle (DMSO 0.1%) or increasing concentrations of the compounds. Cell viability was assessed with AlamarBlue assay. 10% AlamarBlue solution (Thermo Fisher Scientific) was added to the cell culture medium and maintained in 37 °C for 2 h. A SpectraMax M5 fluorescence microplate reader (Molecular Devices, Sunnyvale, CA) was used for collecting the AlamarBlue fluorescence at Ex560/Em590. For cell proliferation assay, the cell images were collected by an IncuCyte ZOOM (Essen BioScience, Ann Arbor, MI). The cell confluences were analyzed for proliferation rate by IncuCyte ZOOM software.

### Colony formation assay

Briefly, the cells were seeded into 6-well plates at a density of 1000 cells/well. After 24 h, cells were treated with Stattic (10 μM) for 48 h, which was then removed and replaced by medium and incubated for 10-14 days. The colonies were fixed with methanol for 15 min, stained with crystal violet at room temperature for 30 min, and then washed and counted.

### Western blotting

The cells were lysed with 2X Laemmli sample buffer (65.8 mM pH6.8 Tris-HCl (Bio-Rad), 26.3% (w/v) glycerol (IBI Scientific, USA), 2.1% SDS (Bio-Rad) and 0.01% bromophenol blue (Sigma)) for protein extraction. The protein lysates were subjected to denaturation by boiling with SDS sample buffer at 95 °C for 5 minutes and subsequently separated using SDS-PAGE. The separated proteins were then transferred onto PVDF membranes, followed by blocking with 5% nonfat dried milk for 1 hour at room temperature. The blots were incubated with the relevant primary antibodies at 4 °C overnight, and then incubated with secondary antibodies for 1 hour at room temperature. Visualization of protein bands was achieved using an enhanced chemiluminescence solution (Thermo Fisher Scientific) and recorded using a ChemiDoc MP imaging system (Bio-Rad, Hercules, CA, USA). Acquisition and analysis of Western blot images were performed using Image Lab software (version 5.1). The information of used primary antibodies and the secondary antibodies were listed in Supplementary Table 1.

### siRNA silencing

The siRNAs used in this study were synthesized by Integrated DNA Technologies (Coralville, IA). In brief, the Lipofectamine RNAiMAX transfection reagent (Thermo Fisher Scientific) and the siRNA were separately diluted in Opti-MEM medium. The Lipofectamine components were then combined and incubated for 5 minutes. Subsequently, cells were seeded into plates containing the transfection mixture. The viability of the cells was evaluated using the AlamarBlue assay, and the efficiency of knockdown was determined through Western blot analysis. The sequence details of the siRNAs are provided in Supplementary Table 2.

### Plasmids and transfection

The plasmids used were transfected with Lipofectamine 3000 reagent (Thermo Fisher Scientific) by the forward transfection method. Briefly, the plasmid and Lipofectamine 3000 was prepared with Opti-MEM (Thermo Fisher Scientific, USA) separately, and then mixed for 5 min. The mixture was dropped into the medium containing cells. After 24 h incubation, cells were treated with compounds or DMSO for 72 h, and then analyzed the cell viability with AlamarBlue assay. The transfection efficiency was confirmed by Western blot assay. pCer-C3-Plk1-T210D was a gift from Dr. Catherine Lindon (Addgene plasmid # 68133).

### Cell cycle and apoptosis analysis by Flow cytometry

For cell cycle analysis: cells were cultured and treated with DMSO or Stattic for 24 h, and then collected, washed with PBS, and fixed in pre-chilled ethanol (Sigma) at -20 °C overnight. Afterward, the fixed cells were rinsed with PBS and stained using a PBS solution containing 100 μg/ml Ribonuclease A (RNase A), 50 μg/ml propidium iodide (PI), and 0.1% Triton X-100 at room temperature for 15 min. For apoptosis analysis: apoptotic cells were quantified using Alexa Fluor 488 annexin V/Dead Cell Apoptosis Kit (Thermo Fisher Scientific). Cells were harvested after treatment, and labeling was performed according to the manufacturer's instructions. Stained cells were analyzed by BD Accuri C6 flow cytometer (BD Biosciences, CA).

### Real-time PCR assay

Total RNA was extracted using Cell Lysis (CL) buffer (10 mM Tris pH7.4 (Invitrogen, USA), 0.25% IGEPAL CA-630 (Sigma), and 150 mM NaCl). cDNAs were synthesized from 1 µg of total RNA using High-Capacity cDNA Reverse Transcription Kit (Thermo Fisher Scientific). The cDNA samples were measured with Real Time PCR. ITaq Universal SYBR Green Supermix (Bio-Rad, USA) was employed to label double-stranded DNA, and the procedure was carried out using the C1000 TouchTM Thermal Cycler (CFX96TM Real-Time System, Bio-Rad). All primers used in this study were purchased from BGI Genomics (Hong Kong), and their sequences can be found in Supplementary Table 3.

### Immunofluorescence assay

Cells were seeded into an 8-well Chambered Coverglass with non-removable wells from Thermo Fisher Scientific. Following a 48-hour treatment, cells were fixed using 4% PFA in PBS for 15 min at room temperature. Subsequently, cells were permeabilized with 0.2% Triton X-100 for 10 minutes and then blocked with 1% BSA and 22.52 mg/mL glycine in PBST (PBS + 0.1% Tween 20) for 30 minutes. The cells were then immuno-stained with primary antibodies, which were diluted in the blocking buffer, and incubated overnight at 4 °C. After this, cells were rinsed with PBS and then treated with Alexa Fluor 488- and 594-conjugated secondary antibodies for 1 hour at room temperature. A DAPI solution was used for nucleus staining for 1 minute. The immunofluorescence images were captured utilizing a Nikon A1R Confocal System.

### *In vivo* xenograft model

The Animal Research Ethics Committee of the University of Macau granted approval for all animal procedures. Female nude mice aged six to eight weeks were subcutaneously implanted with 1 × 10^7^ HCT116 *PTEN^+/+^* and HCT116 *PTEN^-/-^*, DLD1 *PTEN^+/+^* and DLD1 *PTEN^-/-^* or RKO *PTEN^+/+^* and RKO *PTEN^-/-^* cells with Matrigel on two flanks. When the tumor volume reached approximately 100 mm^3^, mice were randomized for grouping. The treatment included vehicle (sterile saline with 5% DMSO, 5% Tween-80, and 5% polyethylene glycol-400) and Stattic (5 and 10 mg/kg, daily) through intraperitoneal injection. Tumor size was periodically measured using calipers, and the tumor volume (mm^3^) was calculated using the ellipsoid formula (volume = L × W^2^ × π/6). The mice's body weight was regularly recorded to monitor drug toxicity. Upon completion of the treatment period, the mice were euthanized, and the tumors were excised, weighed, and preserved in a liquid nitrogen storage tank.

### Patient-derived organoid model

Colon cancer patient samples were obtained from Kiang Wu Hospital in Macau. Ethical approval and the procedures for preparation of patient-derived organoid culture were described previously 13.

### Statistics

Analyses were carried out within the programs GraphPad Prism 6.0. Tests resulting in *p* values less than 0.05 were considered significant. Significance thresholds: *p < 0.05, **p < 0.01. Error bars reflect the standard error of standard deviations (SD). Group comparisons were tested by two-way ANOVA. Unpaired t test was used for one sample test. Western blot data shown were repeated with at least three independent experiments.

## Results

### PTEN-deficient colorectal cancer cells are highly sensitive to STAT3 inhibitor

**Figure 1:**
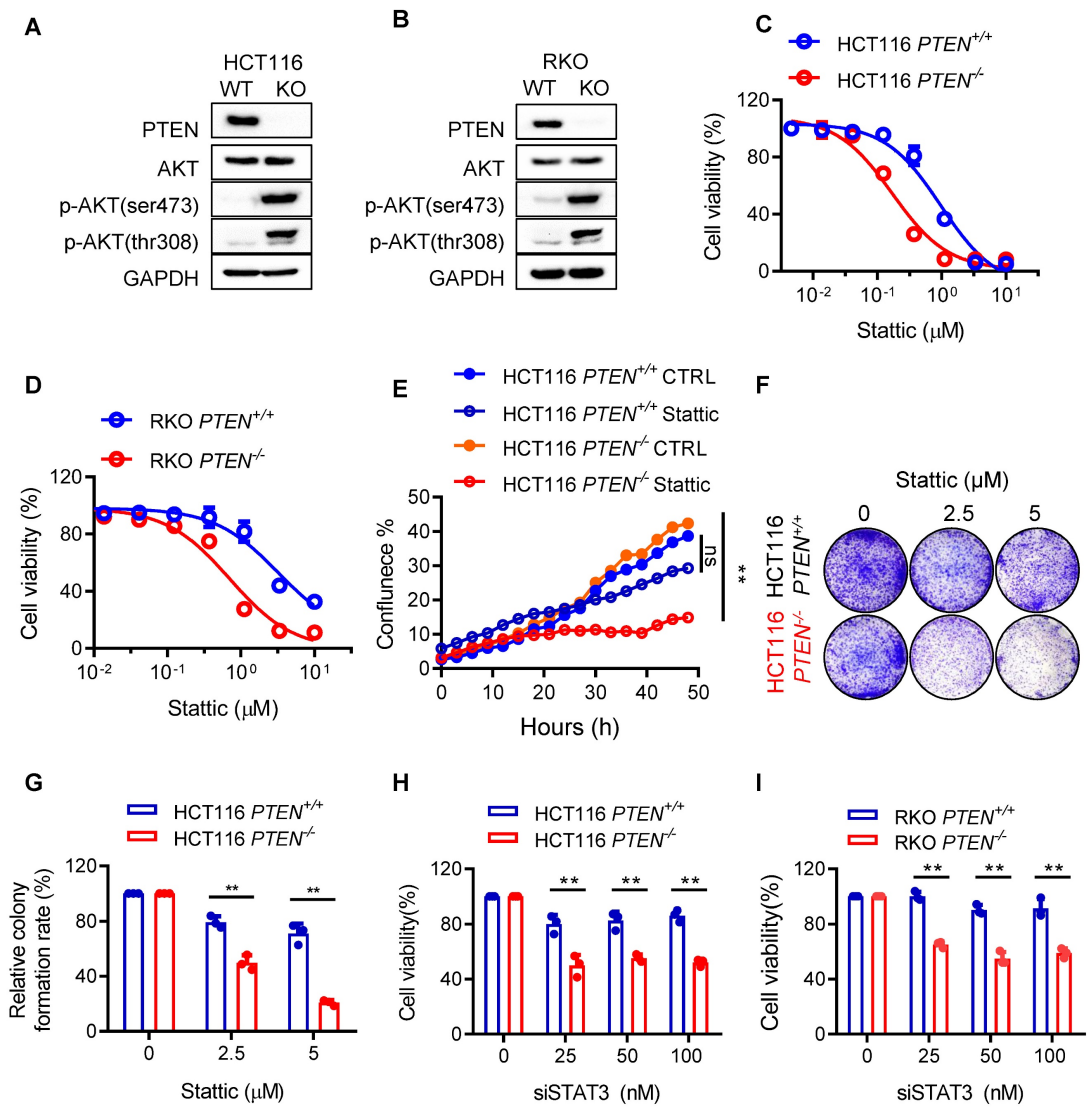
** PTEN is synthetic lethal with STAT3 *in vitro*. (A, B)** Immunoblots showing the protein level of PTEN, AKT, p-AKT(ser473) and p-AKT(thr308) in PTEN-isogenic HCT116 and RKO cells**.** Blots are representative of three independent experiments.** (C, D)** Cell viability analysis of Stattic for 72 h in HCT116 PTEN-isogenic pairs (C) and RKO PTEN-isogenic pairs (D), presented as means ± SD of three independent assays. **(E)** Survival curves for HCT116 PTEN-isogenic pairs treated with 5 μM Stattic for 48 h, ns. not significant, **P < 0.01 between two indicated curves (two-way ANOVA test).** (F)** Colony formation assay of HCT116 PTEN-isogenic pairs with the indicated treatment. **(G)** Quantification of the colony formation. Data are presented as mean ± SD (n = 3 independent experiments), **P < 0.01 between two indicated bars (two-way ANOVA test). **(H, I)** HCT116 PTEN-isogenic pairs (H) and RKO PTEN-isogenic pairs (I) were treated with the indicated concentrations of STAT3 siRNA for 72 h, and cell viability were analyzed. Data are presented as mean ± SD (n = 3 independent experiments), **P < 0.01 between two indicated bars (two-way ANOVA test).

In order to identify the essential target genes for survival of CRC cells in PTEN specific context, we previously established PTEN-isogenic HCT116 and RKO cell lines and screened for synthetic lethal drug candidates in a target-defined small molecule library 12, 13. Among the identified candidates, the STAT3 inhibitor Stattic presented one of the top synthetic lethal drugs in PTEN-deficient CRC cells 13. We thus sought to validate the synthetic lethal effect of Stattic in the PTEN-isogenic HCT116 and RKO cell lines. The PTEN status and its downstream phosphorylated AKT in the PTEN-isogenic pairs were verified with Western blots (Fig. 1A-B). Stattic treatment selectively inhibited the cell viability in *PTEN*^-/-^ HCT116 and RKO cells over the *PTEN*^+/+^ counterparts (Fig. 1C-D). A real-time cell growth analysis with an Incucyte cell imaging also showed that *PTEN*^-/-^ cells were selectively vulnerable to Stattic treatment (Fig. 1E). Finally, a colony formation assay further showed that Stattic selectively suppressed the clonogenic growth of *PTEN*^-/-^ CRC cells over *PTEN*^+/+^ ones (Fig. 1F-G), verifying that Stattic is a synthetic lethal drug for PTEN-deficient CRC cells *in vitro*. To investigate whether the synthetic lethal effect of Stattic is mediated by the inhibition of STAT3 rather than its off-target effect, we silenced STAT3 using specific siRNA and analyzed the synthetic lethal effect in PTEN-deficient CRC cells. Like Stattic treatment, STAT3 silencing also selectively inhibited the growth of PTEN-deficient CRC cells (Fig. 1H-I), indicating that the Stattic-induced synthetic lethality is mediated by the inhibition of STAT3 activity.

### STAT3 inhibition induces polyploidy and mitotic arrest in PTEN-deficient colorectal cancer cells

**Figure 2:**
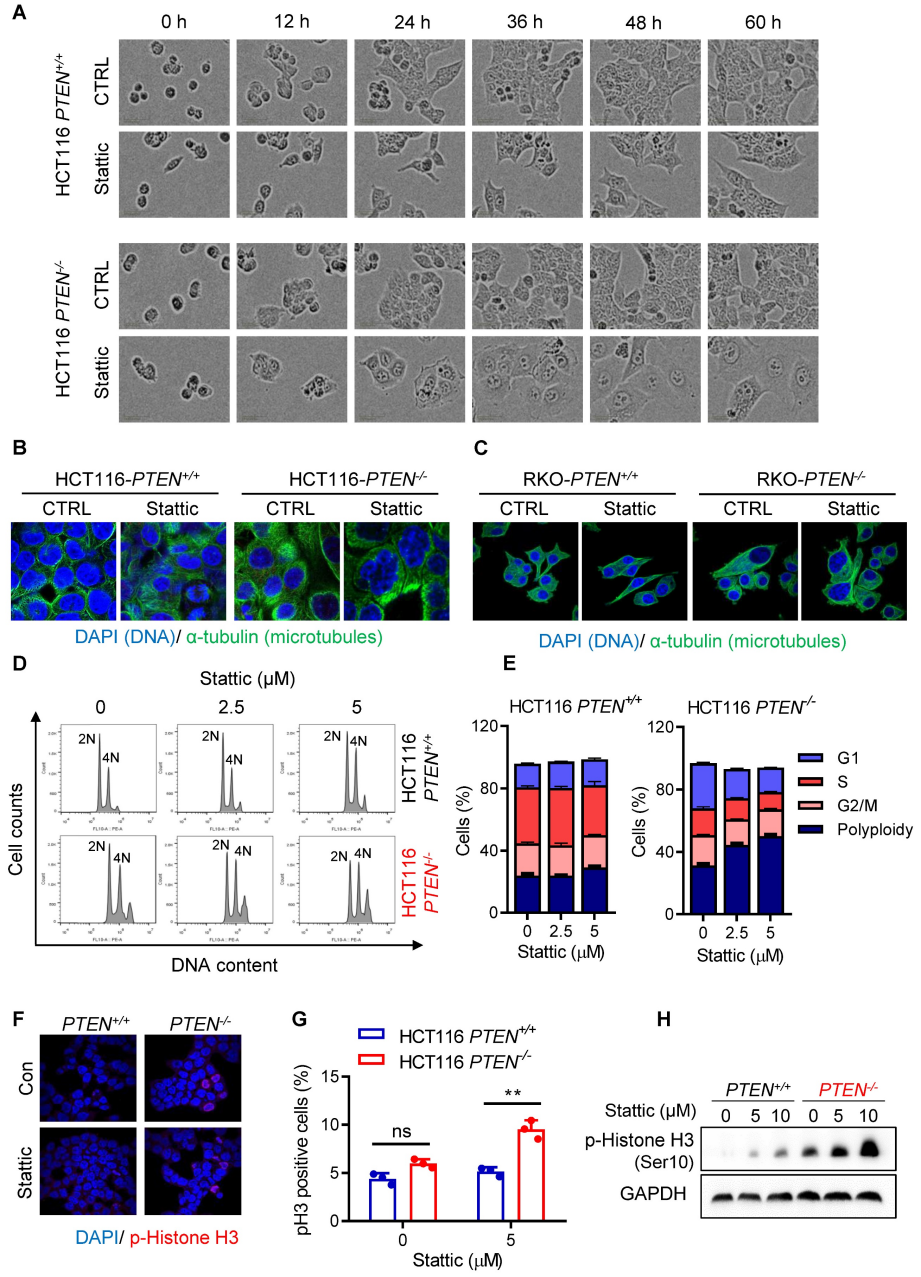
** STAT3 inhibitor induced abnormal cell accumulation and G2/M arrest. (A)** The cell images were respective of HCT116 *PTEN^+/+^* and* PTEN^-/-^* cells with Stattic treatment. **(B, C)** PTEN-isogenic cells were treated with 5 μM Stattic for 24 h, and the cells were processed for immunostaining of α-tubulin and DAPI.** (D)** The cell cycle regulation effect of STAT3 inhibitor on HCT116 *PTEN^+/+^* and* PTEN^-/-^* cells. Cells were treated with the indicated concentrations of Stattic for 48 h and the cell cycle was analyzed by flow cytometry.** (E)** Quantification of cells accumulation in indicated cell cycle phases. Data are presented as mean ± SEM (n = 3 independent experiments). **(F)** HCT116 *PTEN^+/+^* and* PTEN^-/-^* cells were analyzed by immunofluorescence using phospho-histone H3 (Ser10) antibodies (red) and DAPI (blue). **(G)** Phospho-histone H3 positive cells were calculated by taking the ratio of mitotic cells (Phospho-histone H3 positive) and total number of cells (DAPI positive, blue). Data are presented as mean ± SEM (n = 3 independent experiments), **P < 0.01 between two indicated bars (two-way ANOVA test). **(H)** Expression level of p-Histone H3 was examined by immunoblotting.

To explore the mechanism of how the STAT3 inhibitor induced the growth defects in PTEN-deficient cells, we conducted a real-time monitoring of cell growth and morphological changes after treatment with a sublethal concentration of Stattic or DMSO. Control *PTEN*^+/+^ or *PTEN*^-/-^ CRC cells grew normally with increasing the cell population over time. Stattic-treated *PTEN*^+/+^ cells also grew normally, albeit the growth rate was a bit slower than that of control *PTEN*^+/+^ cells. However, Stattic-treated *PTEN*^-/-^ cells did not show an increase in cell number, but the cell and nucleus sizes were significantly enlarged (Fig. 2A for selected images; Supplementary Fig. S1 for full panel of images). Since nuclear enlargement can occur due to various stresses, such as genotoxic stress (e.g., DNA damage) and mitotic stress (e.g. mitotic failure and aneuploidy), we further analyzed the cell and nuclear structures using α-tubulin and DAPI immunostaining. The result showed that a majority of *PTEN*^-/-^ cells exhibited a multi-nuclear, giant cell morphology following Stattic treatment (Fig. 2B-C), suggesting that the compound affects the mitotic process. To further investigate the effect of STAT3 inhibitor on mitotic process, we analyzed the cell cycle and phosphorylated histone H3 (p-HH3) staining, a mitotic cell marker 14, in PTEN-isogenic cell pair treated with or without Stattic. Similar to what we observed in cell morphology, Stattic significantly increased the G2/M population in *PTEN*^-/-^ cells with an increase in the cell population of DNA content greater than 4N, an indicative of cell polyploidy (Fig. 2D-E; Supplementary Fig. S2). Interestingly, untreated PTEN-deficient cells also showed an increase in cell polyploidy compared to control PTEN-wildtype cells (Fig. 2D; Supplementary Fig. S2). We therefore assumed that PTEN-deficient cells have a defect in mitotic quality control, causing increased polyploidy, and that Stattic exacerbates the mitotic abnormalities in these cells. In addition, Stattic treatment significantly increased the number of p-HH3-positive cells in *PTEN*^-/-^ cells (Fig. 2F-H). These results suggest that STAT3 inhibitor induces a selective mitotic failure in PTEN-deficient colorectal cancer cells, causing mitotic arrest and increased polyploidy.

### STAT3 inhibition exacerbates mitotic abnormalities and CIN in PTEN-deficient cells, leading to apoptotic cell death

**Figure 3:**
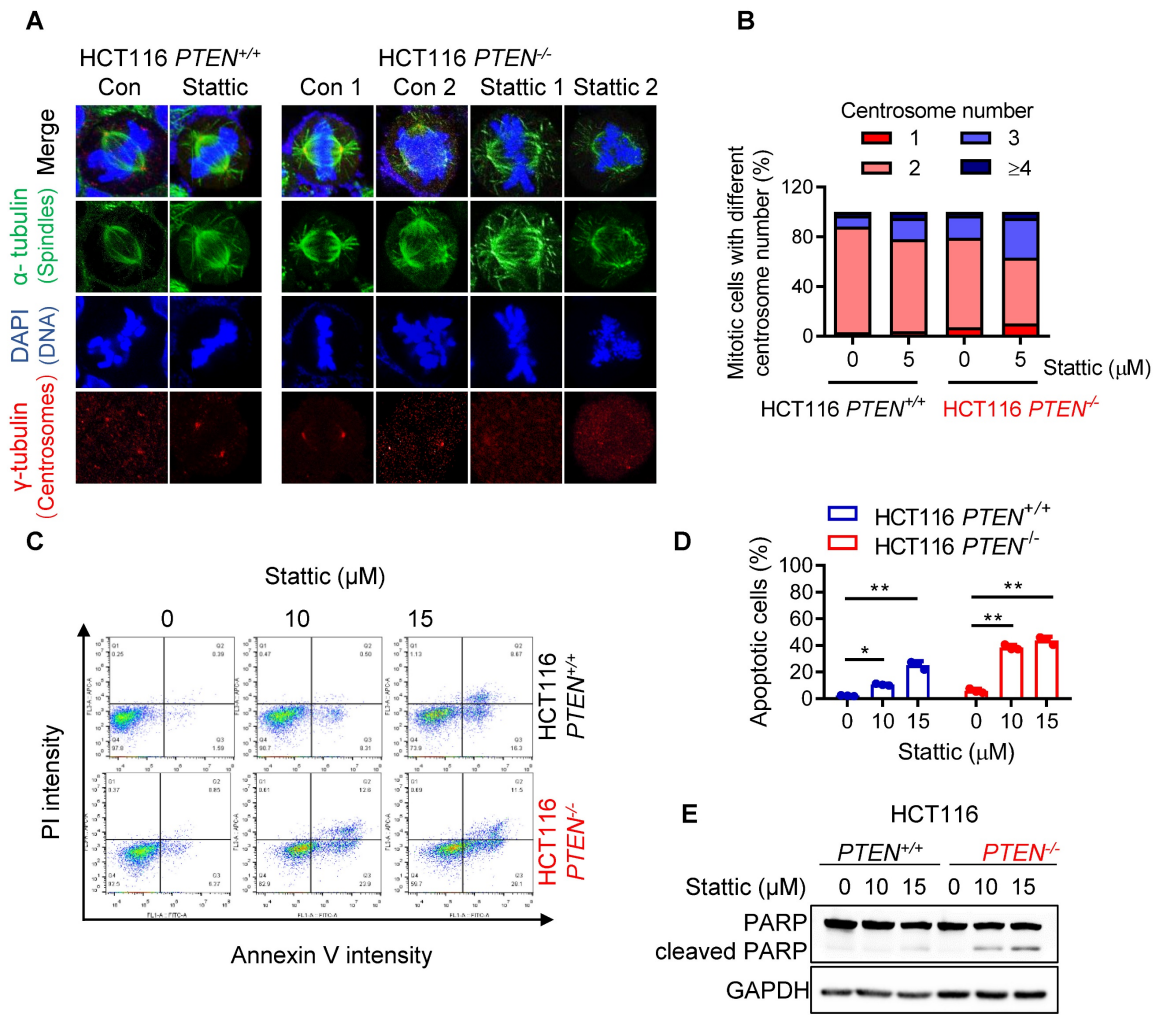
**STAT3 inhibitor induced abnormal spindle and mitotic defect in *PTEN^-/-^* cells. (A)** HCT116 *PTEN^+/+^* and* PTEN^-/-^* cells were treated with 5 μM Stattic for 24 h. The spindle and mitotic DNA were analyzed with the immunofluorescence staining of α-tubulin (green), γ-tubulin (red), and DNA (blue). Scale bars, 10 μm. **(B)** The percentage of mitotic cells with the number of centrosomes was obtained from the images of γ-tubulin spots. **(C, D)** Effect of STAT3 inhibitor on apoptosis in HCT116 *PTEN^+/+^* and* PTEN^-/-^* cells. Cells were treated with 10 and 15 μM Stattic for 48 h and then measured with Annexin V-FITC/propidium iodide staining (C). Quantification of apoptotic cells (D). Data are presented as mean ± SD (n = 3 independent experiments), **P < 0.01 between two indicated bars (two-way ANOVA test). **(E)** Cell apoptosis was measured with the Western blots of PARP and cleaved PARP.

To investigate the effect of STAT3 inhibitor on the mitotic process in greater detail, we analyzed the metaphase cells for mitotic spindles and centrosome abnormality using α- and γ-tubulin immunostainings. Control PTEN-deficient cells showed an increase in spindle abnormalities, such as increased multipolar or monopolar spindles with altered centrosome numbers, compared to control PTEN-wildtype cells (Fig. 3A-B). Stattic treatment significantly increased the number of cells with abnormal spindles in *PTEN*^-/-^ cells (Fig. 3A-B). This phenomenon is similar to cell polyploidy phenotype where Stattic exacerbated the chromosomal abnormality in PTEN-deficient CRC cells. Therefore, we further assumed that PTEN-deficient cells have a defect in mitotic quality control, causing mitotic abnormalities and chromosomal instability, making the cells more sensitive to Stattic treatment. Next, to investigate the cell fate following STAT3 inhibitor-induced mitotic abnormalities, we analyzed apoptosis markers in cells treated with or without Stattic. Both the annexin V/PI staining and PARP cleavage assay demonstrated that Stattic selectively induced apoptotic cell death in *PTEN*^-/-^ CRC cells (Fig. 3C-E). These results indicate that PTEN-deficient CRC cells have an intrinsic deficiency in mitotic quality control, leading to abnormal mitotic phenotypes and CIN. These phenotypes render the cells hypersensitive to STAT3 inhibition.

### PTEN deficiency creates cellular dependency on PLK1 activity

**Figure 4:**
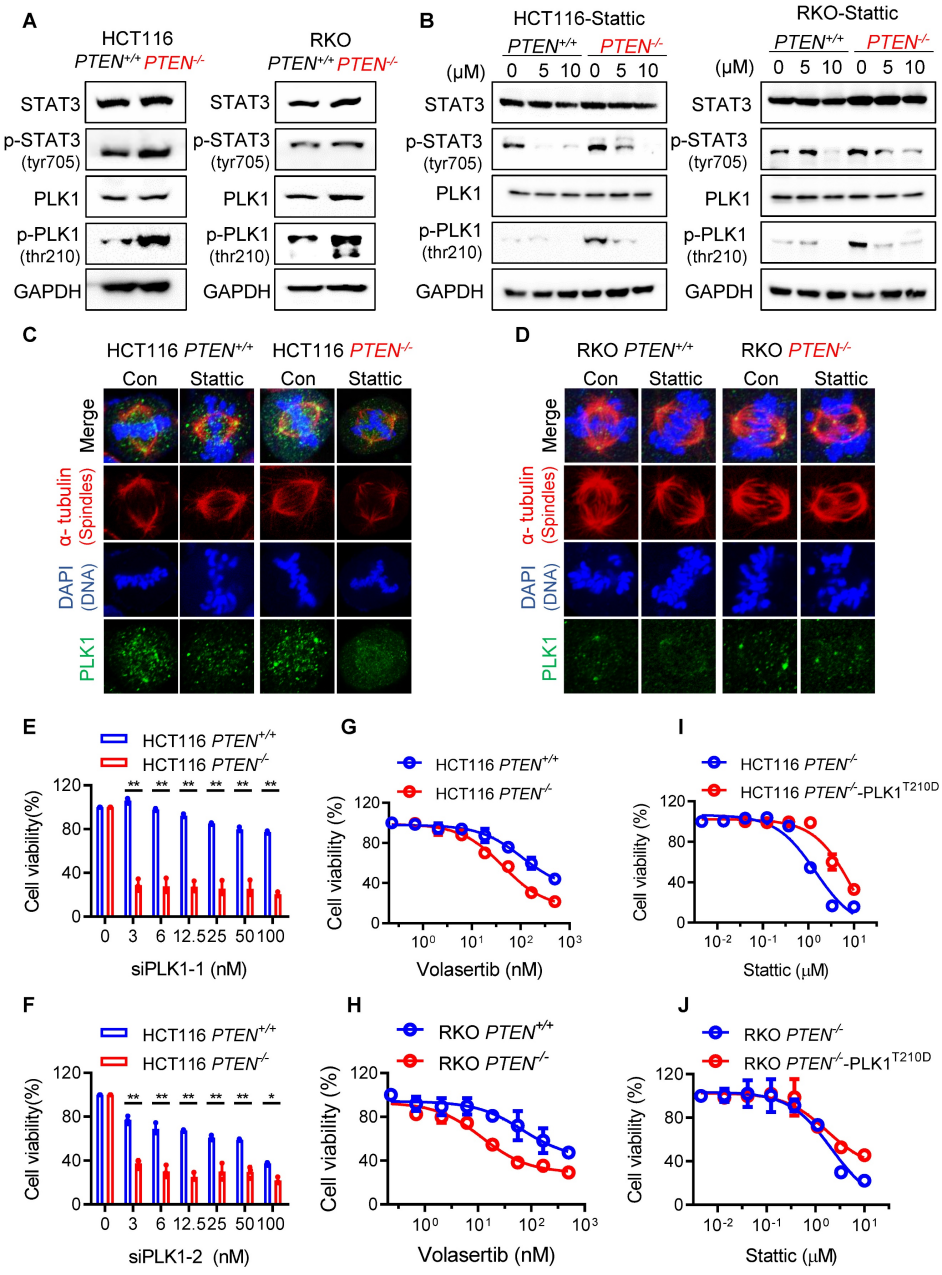
**PLK1 mediated the synthetic lethality of PTEN and STAT3. (A)** Immunoblots showing total STAT3 and PLK1 levels and their phosphorylation levels in PTEN-isogenic HCT116 and RKO cells. **(B)** Cells were treated with Stattic for 24 h and the western blot analyses of STAT3, p-STAT3, PLK1 and p-PLK1.** (C, D)** Immunofluorescence images of α-tubulin (red), PLK1 (green) and DAPI (blue) in mitotic HCT116 *PTEN^+/+^* and* PTEN^-/-^* cells (C) or RKO *PTEN^+/+^* and* PTEN^-/-^* cells (D) treated with 5 μM Stattic. **(E, F)** Viability analysis in PLK1 knockdown HCT116 PTEN-isogenic cells with two different siRNA. Data are presented as mean ± SD (n = 3 independent experiments), *P < 0.05, **P < 0.01 between two indicated bars (two-way ANOVA test). **(G, H)** Viability analysis in HCT116 (G) and RKO (H) PTEN-isogenic cells with PLK1 inhibitor Volasertib treatment. **(I, J)** Viability analysis in HCT116 *PTEN^-/-^* cells (I) and RKO *PTEN^-/-^* cells (J) with transiently overexpressing PLK1 T210D and treated with Stattic for 72 h. Data are presented as mean ± SD (n = 3 independent experiments).

Since we observed that PTEN deficiency led to a deficiency in mitotic quality control and STAT3 inhibition exacerbated the abnormal mitotic phenotypes, we sought to find a common factor for the abnormal mitotic phenotypes in both pathways. From a thorough literature study, Polo-like kinase 1 (PLK1) appeared as the best candidate mediator because it is a substrate of PTEN phosphatase 6 involved in the chromosomal stability as well as a downstream target of STAT3 signal involved in the centrosome clustering 15. We thus analyzed STAT3 and PLK1 signaling activities in PTEN-isogenic CRC pair. The levels of phosphorylated STAT3 and PLK1 were elevated in* PTEN*^-/-^ cells (Fig. 4A). Inhibition of STAT3 by Stattic not only suppressed STAT3 phosphorylation, but also reduced PLK1 phosphorylation (Fig. 4B). The inhibitory effect was much more pronounced in *PTEN*^-/-^ cells where phosphorylated STAT3 and PLK1 were highly elevated. These data suggest that STAT3 signal is activated in PTEN-deficient cells and acts as an upstream activator of PLK1. Immunostaining of PLK1 in mitotic cells showed that centrosomal PLK1 localization is strong and clear in *PTEN*^-/-^ cells, while Stattic treatment significantly weakened its centrosomal localization with disturbed normal centrosome separation (Fig. 4C-D). Considering the essential role of PLK1 in the mitotic process, our findings indicate that the STAT3-PLK1 pathway is hyperactivated in PTEN-deficient cells, which may contribute to mitotic abnormalities and CIN. The STAT3 pathway is known to be positively regulated by mTOR signaling, while PTEN functions as a negative regulator of both STAT3 and mTOR pathways in other cancer types 16. We therefore investigated the upstream regulation of STAT3 in PTEN-deficient CRC cells. Treatment of PTEN-deficient CRC cells with LY294002 (PI3K/mTOR inhibitor) and rapamycin (mTOR inhibitor) inhibited phosphorylation of AKT and mTOR, while Stattic did not. However, all three inhibitors—LY294002, rapamycin, and Stattic—effectively suppressed STAT3 phosphorylation in the cells (Supplementary Figure S3). These findings suggest that the PI3K/mTOR/AKT pathway acts as an upstream regulator of STAT3 activation in PTEN-deficient CRC cells. To further investigate the role of PLK1 in the synthetic lethal relationship between PTEN and STAT3, we analyzed the synthetic lethal effect of PLK1 inhibition in PTEN-isogenic CRC cells. The depletion of PLK1 by two different PLK1 siRNAs (siPLK1-1 and siPLK1-2) showed that both siRNA induced synthetic lethality in *PTEN*^-/-^ cells (Fig. 4E-F; Supplementary Fig. S4A-B). Moreover, the PLK1 specific inhibitor volasertib also induced synthetic lethality in *PTEN*^-/-^ cells (Fig. 4G-H). Overexpression of PLK1^T210D^, a constitutively active form of PLK1, partly rescued the synthetic lethal effect of Stattic in *PTEN*^-/-^ cells (Fig. 4I-J; Supplementary Fig. S5A-B). Altogether, these data suggest that PTEN deficiency activates STAT3 signal and PLK1 activity in cells, and this in turn creates the cellular dependency on PLK1 activity for mitotic progression and survival.

### STAT3 inhibition depends on stathmin (STMN1) to suppress PLK1 phosphorylation and promote cell death in PTEN-deficient CRC cells

**Figure 5:**
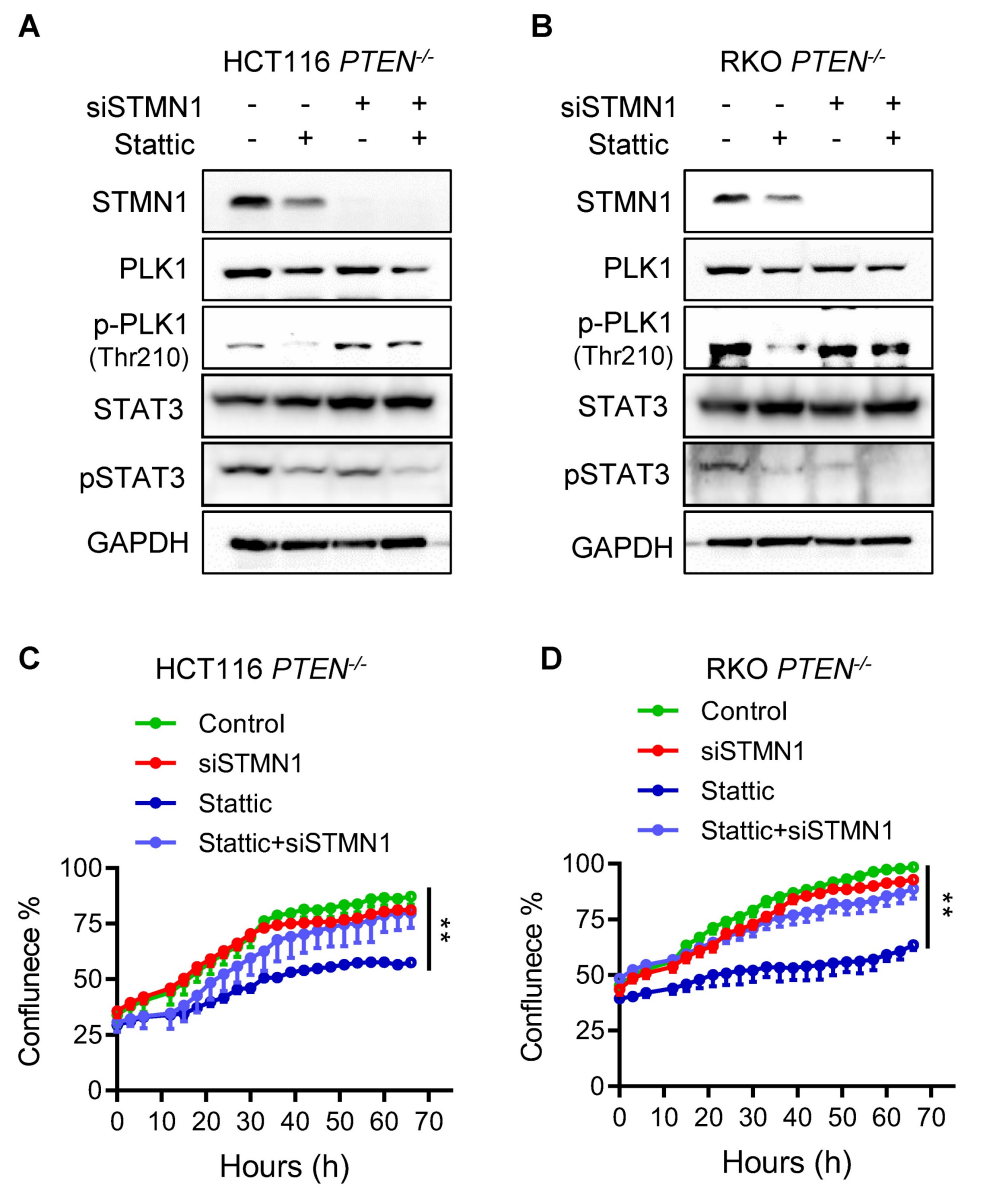
** STAT3 inhibitor suppressed PLK1 via regulating stathmin. (A, B)** Immunoblot showing STMN1 knockdown efficiency and PLK1 phosphorylation level after STMN1 silencing in HCT116 *PTEN^-/-^* cells (A) and RKO *PTEN^-/-^* cells (B). **(C, D)** Cell growth inhibition effects of Stattic after STMN1 knockdown in HCT116* PTEN^-/-^* cells (C) and RKO *PTEN^-/-^* cells (D). Data are presented as mean ± SD (n = 3 independent experiments), **P < 0.01 between two indicated curves (two-way ANOVA test).

STAT3 is known to act as a transcription activator of PLK1 as it promotes PLK1 transcription by directly binding to PLK1 promoter 17. JAK/STAT3 inhibitors, such as Stattic and AG490, significantly inhibited the expression of PLK1 in esophageal and glioblastoma cancer cells 17, 18. However, in CRC cells Stattic showed very marginal or no effect on the PLK1 mRNA expression level, while it strongly inhibited the PLK1 phosphorylation (Fig. 4B; Supplementary Fig. S6), suggesting that STAT3 acts primarily as an upstream signal to regulate PLK1 activity rather than PLK1 expression in CRC cells. Recent studies suggest that STAT3 regulates PLK1 in a transcription-independent mechanism in which the microtubule destabilizer stathmin (STMN1) is involved. Morris et al. demonstrated that STAT3 directly interacts with STMN1, and this interaction is diminished upon treatment with Stattic. Furthermore, *in vitro* kinase assays using purified proteins revealed that STMN1 suppresses PLK1 kinase activity, while the addition of STAT3 restores PLK1 activity. Stattic treatment again reduced PLK1 activity under these conditions. These findings support a model in which STMN1 negatively regulates PLK1, and STAT3 counteracts STMN1 to activate PLK1 15. STAT3 is also known to regulate microtubule stability via STMN1 where the authors showed that STAT3 directly binds to STMN1 and antagonizes its microtubule-depolymerizing function 19. To explore the possibility that the STAT3-induced PLK1 activation in PTEN-deficient CRC cells was regulated by STMN1, we depleted the STMN1 level using siRNA and analyzed rescue effects on PLK1 activity and cell viability. We observed that Stattic treatment strongly reduced PLK1 phosphorylation along with the reduction in STAT3 phosphorylation in *PTEN*^-/-^ HCT116 cells, whereas this effect was significantly rescued when STMN1 was depleted (Fig. 5A). Same result was observed in *PTEN*^-/-^ RKO cells (Fig. 5B). Interestingly, Stattic treatment also reduced the protein level of STMN1, implying an existence of possible feedback-loop between STAT3 and STMN1 regulation. Our study with mRNA and protein stability regulation of STMN1 suggests that STAT3 positively regulates STMN1 expression at the transcriptional level (Supplementary Fig. S7A-C). Interestingly, we observed that depletion of STMN1 led to a reduction in phosphorylated STAT3 levels (Fig. 5A-B). This finding suggests a potential reciprocal regulatory relationship between STAT3 and STMN1, possibly mediated through their physical interaction and complex formation. Further investigation is warranted to elucidate the mechanisms underlying this interplay. Additionally, STMN1 depletion itself had no effect on the cell viability, while it significantly rescued the Stattic-induced cell death in *PTEN*^-/-^ cells (Fig. 5C-D). These results suggest that STAT3 inhibition suppresses PLK1 phosphorylation and promotes cell death in PTEN-deficient CRC cells through a mechanism that depends on STMN1. In addition, our results indicate that STAT3 acts as an upstream activator of PLK1 activity rather than its expression in PTEN-deficient CRC cells.

### The mitotic cell death induced by STAT3 inhibitor in PTEN-deficient CRC cells is mediated by spindle assembly checkpoint (SAC) hyperactivation

**Figure 6:**
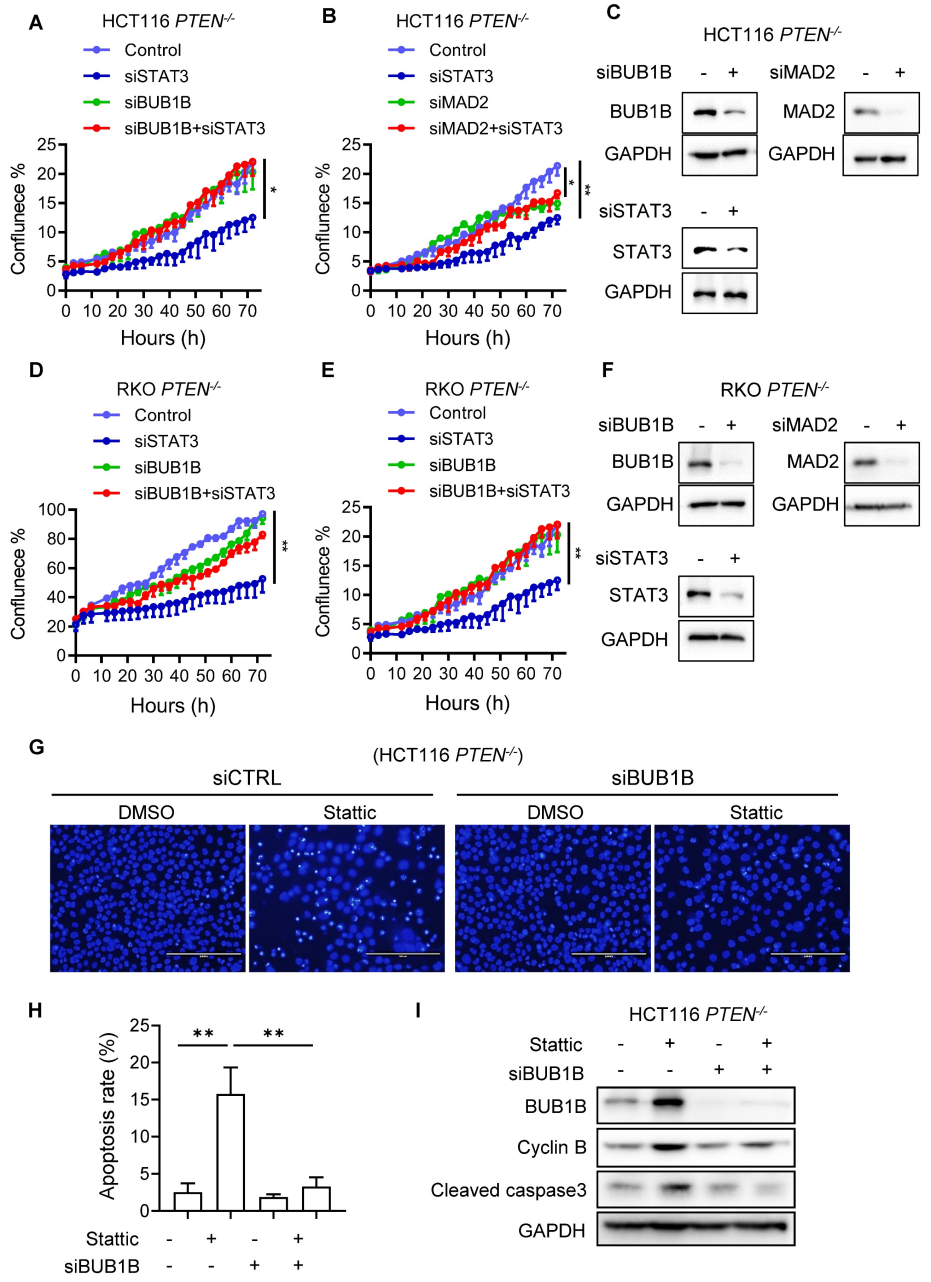
** STAT3 inhibitor induced spindle assembly checkpoint (SAC) activation in PTEN^-/-^ cells. (A, D)** Effect of BUB1B siRNA on the cell sensitivity of HCT116 *PTEN^-/-^* cells (A) and RKO *PTEN^-/-^* cells (D) to STAT3 inhibition.** (B, E)** The effect of MAD2 siRNA on the cell sensitivity of HCT116 *PTEN^-/-^* cells (B) and RKO *PTEN^-/-^* cells (E) to STAT3 inhibition. Data are presented as mean ± SD (n = 3 independent experiments), *P < 0.05, **P < 0.01 by two-way ANOVA test. **(C, F)** The BUB1B, MAD2 and STAT3 silencing efficiency were examined by Western blots.** (G)** Effect of control and BUB1B siRNA on Stattic-induced apoptosis in HCT116 *PTEN^-/-^* cells. Cell apoptosis was evaluated by observing nuclear fragmentation and chromatin condensation following Hoechst 33342 staining. **(H)** Quantitation of the apoptotic cells stained with Hoechst 33342. **P < 0.01 between two indicated bars (Student's *t*-test). I. Effect of BUB1B siRNA on Stattic-induced SAC activation and apoptosis in HCT116 *PTEN^-/-^* cells. SAC activation was evaluated with BUB1B and Cyclin B level, and apoptosis was evaluated with the level of cleaved caspase-3.

We earlier found that STAT3 inhibition led to a severe abnormal spindle formation, polyploidy, mitotic arrest and death in PTEN-deficient cells. The spindle assembly checkpoint (SAC), also known as the mitotic checkpoint, is a crucial quality control mechanism that ensures accurate chromosome segregation during cell division. It monitors the attachment of chromosomes to the mitotic spindle and delays the onset of anaphase until all chromosomes are properly aligned and attached 20. During mitosis phases, PLK1 localizes to kinetochores and regulates the attachment of kinetochores and spindle microtubules, while PLK1 inhibition results in mitotic arrest due to SAC activation caused by defects in kinetochore-microtubule attachments 21, 22. When the severity of spindle abnormalities is beyond the cell's repair capacity, the SAC will trigger a prolonged mitotic arrest, often leading to cell death to maintain genomic stability. Given that PTEN-deficient CRC cells exhibit a CIN phenotype due to mitotic abnormalities, and STAT3 inhibition further exacerbates spindle abnormalities and CIN in these cells, SAC hyperactivation is anticipated as a mechanism leading to mitotic arrest and cell death. To test this notion and investigate the cell death mechanism by STAT3 inhibition, we performed rescue experiments by silencing two key components of SAC machinery, BUB1B and MAD2. Depletion of either BUB1B or MAD2 using specific siRNAs significantly rescued the cell growth inhibition induced by STAT3 silencing in PTEN-deficient HCT116 and RKO cells (Figure 6 A-F). To further demonstrate mitotic cell death and SAC activation induced by STAT3 inhibition, we analyzed cell death markers and SAC activity in PTEN-deficient CRC cells treated with either Stattic or BUB1B siRNA, alone or in combination. Treatment with Stattic significantly increased apoptotic cell death in *PTEN*^-/-^ CRC cells, as indicated by nuclear condensation and fragmentation (Fig. 6G-H) and cleaved caspase-3 levels (Fig. 6I). Notably, these apoptotic phenotypes were markedly attenuated by BUB1B silencing. Furthermore, both BUB1B and cyclin B levels were substantially elevated following Stattic treatment, and these increases were reversed to near control levels upon BUB1B silencing (Fig. 6I). BUB1B is a core SAC component that inhibits the anaphase-promoting complex/cyclosome (APC/C), thereby preventing premature mitotic progression. Cyclin B, in complex with CDK1, drives mitotic entry and progression. Under normal conditions, SAC silencing leads to APC/C activation, cyclin B degradation, and anaphase onset. However, when SAC is activated, APC/C is inhibited, resulting in cyclin B accumulation. Thus, elevated cyclin B levels serve as a marker of SAC activation. Our data suggest that Stattic treatment activates the SAC in PTEN-deficient CRC cells, leading to mitotic arrest and cell death. This effect is rescued by BUB1B silencing, supporting the role of SAC hyperactivation in Stattic-induced mitotic death.

### STAT3 inhibition induced synthetic lethality with PTEN deficiency in mice and patient-derived organoids (PDOs)

**Figure 7:**
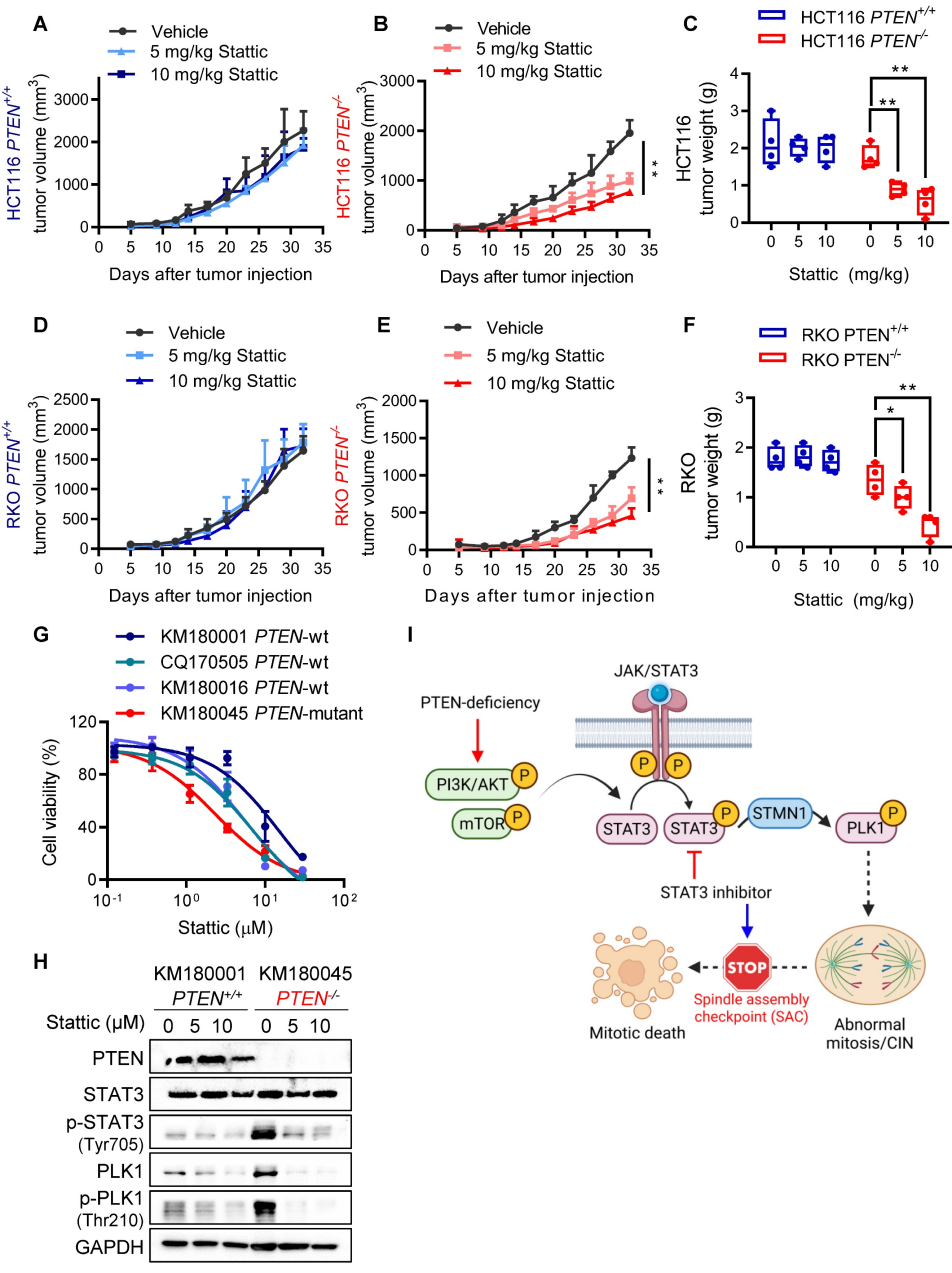
** STAT3 inhibition is synthetic lethal with PTEN deficiency *in vivo*. (A-F)** Tumor growth curves in nude mice bearing HCT116 *PTEN^+/+^*(A) or* PTEN^ -/-^* tumor (B) and RKO *PTEN^+/+^*(D) or* PTEN^ -/-^* tumor (E) with vehicle or 5 and 10 mg/kg Stattic treatment. Wet tumor weights of HCT116 *PTEN^+/+^* or *PTEN^ -/-^* tumors (C) and RKO *PTEN^+/+^* or *PTEN^ -/-^* (F) at endpoint. Error bars represent s.d. *P < 0.05, **P < 0.01 between vehicle and Stattic treatment groups (n = 4), two-way ANOVA. **(G)** Viability of patient-derived colorectal tumor organoids (PDOs) with 48 h Stattic treatment. **(H)** Immunoblot showing PTEN, STAT3, p-STAT3, PLK1, p-PLK1 and STMN1 protein levels in PDOs. I. Schematic illustration of the mechanism of synthetic lethality between PTEN and STAT3. STAT3 signal is activated in PTEN-deficient cells by activated PI3K/AKT/mTOR pathway. Activated STAT3 stimulates PLK1 signal via inhibiting STMN1, leading to abnormal mitosis and CIN. Inhibition of STAT3 in these cells promotes mitotic cell death via activating SAC.

To validate the synthetic lethality of PTEN and STAT3 *in vivo*, mice bilateral xenograft models with HCT116 and RKO PTEN-isogenic cell pairs were established and tested with vehicle or two doses of Stattic (5 and 10 mg/kg). Consistent with *in vitro* results, PTEN-deficient tumors were significantly more sensitive to Stattic than PTEN-wildtype tumors, evidenced by reduction in tumor volumes and wet tumor weights (Figure 7 A-F). To further strengthen our findings, we generated an additional PTEN-knockout (KO) CRC cell line (DLD1, originally PTEN wild-type) using the CRISPR/Cas9 system (Supplementary Fig. S8A). We then evaluated the effects of STAT3 inhibition both *in vitro* and *in vivo*. Our results showed that Stattic selectively inhibited the growth of DLD1 PTEN-KO cells compared to their PTEN-WT counterparts (Supplementary Fig. S8B). In mouse xenograft models, Stattic (10 mg/kg) significantly suppressed tumor growth in DLD1 PTEN-KO xenografts, while having no effect on DLD1 PTEN-WT tumors (Supplementary Fig. S8C-E). Importantly, this dose of Stattic did not induce any observable toxicity in mice (Supplementary Fig. S8F). Additionally, we established 4 patient-derived organoids (PDO) from colorectal cancer patient samples, identified to be 3 PTEN-wildtype and 1 PTEN-mutant tumors and tested the effect of Stattic on the growth of the PDO culture. Stattic exhibited stronger inhibitory effect on PTEN-mutant PDOs compared to PTEN-wildtype ones (Fig. 7G). We analyzed PTEN and STAT3 pathway status in the two representative PDOs (PTEN-wildtype and PTEN-mutant) cultures. Indeed, PTEN-mutant PDOs exhibited highly increased STAT3 phosphorylation and PLK1 phosphorylation compared to PTEN-wildtype PDOs, and Stattic strongly inhibited the STAT3-PLK1 signaling in PTEN-mutant PDOs (Fig. 7H). Collectively, these results demonstrated that the STAT inhibition elicited synthetic lethality with PTEN deficiency *in vivo* and patients' samples.

## Discussion

The intricate relationship between genomic and chromosomal instability and cancer progression has unveiled new avenues for developing therapeutic strategies for cancer treatment. CIN is one such feature of CRC with more than half of all sporadic CRCs being of the CIN type. In this study, we found that PTEN-deficient CRC exhibited CIN phenotypes and this feature made the cells hypersensitive to STAT3-PLK1 inhibitors. Our mechanistic study suggests that PTEN deficiency promotes STAT3-PLK1 over-activation, thereby promoting mitotic spindle abnormality and CIN phenotypes. Inhibition of STAT3 or PLK1 exacerbates the mitotic abnormality and CIN in PTEN-deficient CRC, inducing SAC-dependent mitotic cell death (summarized in Fig. 7I). These phenomena were observed in mouse xenograft and PDO models, demonstrating that the STAT3-PLK1 axis could be a potential drug target for the treatment of PTEN-deficient CRC.

The role of PTEN in safeguarding against CIN, beyond its function in blocking the PI3K/AKT pathway, is garnering notable interest. Loss of PTEN is significantly associated with high level of CIN in triple-negative breast cancer 23, ovarian carcinoma 24, endometrial carcinomas 11 and colorectal cancer 11, 25. PTEN was reported to preserve spindle architecture and movement through interaction with CENP-C, PLK1 and EG5 7, 8. Aberrant spindle assembly and segregation is frequently observed in PTEN-deficient cancers. Similarly, we also found an increasing trend of defective spindle architecture and centrosomal integrity following over-activation of PLK1 signal in *PTEN*^-/-^ CRC cells. Given that PLK1 phosphorylation is known to be regulated by PTEN 6, it can be postulated that PLK1 over-activation in PTEN-deficient cells is due to the direct regulation of its phosphorylation by PTEN phosphatase. However, our data show that PLK1 over-activation was mediated by STAT3-STMN1 regulatory axis in PTEN-deficient CRC cells. Inhibition of STAT3 activity strongly suppressed PLK1 phosphorylation in *PTEN*^-/-^ CRC cells and the overexpression of PLK1^T210D^, a phosphomimetic mutant version of PLK1 conferred resistance to Stattic-induced synthetic lethality in *PTEN*^-/-^ CRC cells. Furthermore, STMN1 depletion rescued the inhibition of PLK1 phosphorylation by Stattic, followed by reversing synthetic lethal effect of Stattic in *PTEN*^-/-^ CRC cells. Altogether, these data indicate that STAT3-STMN1 pathway is a primary regulatory upstream of PLK1 activity in PTEN-deficient CRC cells. This also provides a scientific basis for STAT3 as a druggable target for PTEN-deficient CRC in addition to PLK1.

STAT3 is a DNA-binding transcription factor that plays a pivotal role in various cellular processes, including cell growth, survival and immune responses. STAT3 is frequently found to be dysregulated in various cancers, making it a potential therapeutic target 26. It belongs to the signal transducer and activator of transcription (STAT) family of proteins that transduces signals from cytokines and growth factors to intracellular targets. These proteins are usually activated by membrane receptor-associated Janus kinases (JAK) and then translocate to the nucleus to regulate gene expression. STAT3 is activated by cytokines (e.g., IL-6 and IFN-α), growth factor receptors (e.g., EGFR, HER2, and PDGFR), and non-receptor tyrosine kinases (e.g., SRC and all JAK family proteins) through phosphorylation at tyrosine-705 site 27. Following activation, STAT3 forms dimers and translocates to the nucleus and binds to the interferon-gamma activated sequence (GAS) within target gene promoters to modulate gene transcription 26. While STAT3 is traditionally associated with its transcriptional activities, emerging evidence suggests its involvement in mitosis through non-transcriptional mechanisms. STAT3 was reported to physically associate with STMN1 and regulate the stability of microtubules 19. Recent studies highlighted the role of STAT3 in mitotic regulation via modulating STMN1-controlled PLK1 activity during mitosis 15. Our results also suggest that STAT3 inhibition suppressed PLK1 activity through STMN1, thereby disrupting bipolar spindle architecture and mitotic progression in PTEN-deficient CRC cells. This caused SAC-mediated mitotic cell death.

We observed that STAT3 is activated in PTEN-deficient CRC cells, but the mechanism how PTEN loss activates STAT3 in CRC remains elusive. A similar phenotype was observed in pancreatic cancer where loss of PTEN in pancreatic cancer-associated fibroblasts (CAFs) results in the activation of STAT3^28^. In the study, JAK1 inhibitor successfully prevented the STAT3 phosphorylation by PTEN loss, but PI3K inhibitor did not, suggesting that PTEN loss-induced STAT3 activation involves canonical JAK signaling, but not canonical PI3K signaling. Another study showed that the STAT3 pathway is positively regulated by mTOR signaling, while PTEN functions as a negative regulator of both STAT3 and mTOR pathways in breast cancer 16. Our study with the specific pathway inhibitors in CRC cells suggested that the PI3K/mTOR/AKT pathway acts as an upstream regulator of STAT3 activation in PTEN-deficient CRC cells. However, opposite results were also reported recently. de la Iglesia et al., and Wang et al., recently showed that PTEN deficiency inhibited STAT3 signal in brain tumor 29 and gastric cancer 30, respectively. Therefore, there is a strong relationship between PTEN and STAT3 signaling. However, whether this relationship is positive or negative may depend on the type of cancer. Further study is needed to clarify the association between PTEN function and STAT3 signaling in CRC cells.

The role of STAT3 as a central oncogenic driver in CRC is well-established. STAT3 is frequently hyperactivated in CRC and contributes to tumor proliferation, angiogenesis, invasion, immune evasion, and resistance to therapy 31. Our findings align with these findings, reinforcing the therapeutic potential of targeting STAT3, particularly in PTEN-deficient CRC, where STAT3 appears to play a critical role in activation of oncogenic signaling. Several studies have explored STAT3 inhibition in CRC using small molecules such as Stattic, napabucasin, and bruceantinol, showing promising anti-tumor effects in preclinical and clinical models 32, 33. However, despite these advances, STAT3 inhibitors have not yet reached clinical use, largely due to challenges in drug specificity, bioavailability, and toxicity. Importantly, resistance mechanisms to STAT3 inhibition have begun to emerge. For example, compensatory activation of parallel pathways, such as MAPK/ERK or PI3K/AKT, may limit the efficacy of STAT3-targeted therapies 33. These findings underscore the need for combination strategies, such as pairing STAT3 inhibitors with MEK or PI3K inhibitors, to overcome resistance and enhance therapeutic outcomes. Targeting STAT3 in CRC holds promise, but it also raises concerns about off-target effects and toxicity in normal cells, given STAT3's role in many physiological processes, including immune regulation, neuroprotection and hematopoiesis. In a lung cancer model, the STAT3 inhibitor STX-0119 caused a marked reduction in white blood cell counts, indicating potential immunosuppression and hematologic toxicity 34. Additionally, STAT3 inhibition in dendritic cells has been shown to suppress STAT5 signaling, impairing T cell priming and diminishing anti-tumor immune responses 35. To mitigate these adverse effects, combination therapies or synthetic lethal approaches that allow for reduced dosing of STAT3 inhibitors may be considered as strategies to enhance therapeutic efficacy while minimizing toxicity. In light of these findings, our study contributes to the growing evidence that targeting STAT3 in genetically defined CRC subtypes, such as PTEN-deficient tumors, may offer a more precise and effective therapeutic approach.

In conclusion, this study showed a synthetic lethal interaction between PTEN and STAT3, which involves PLK1-mediated mitotic abnormality and CIN in CRC cells. Our study revealed the significance of inhibiting STAT3, as well as PLK1, as a potential therapeutic strategy for treating PTEN-deficient CRC.

## Supplementary Material

Supplementary figures and tables.
