# Supplementary material for: PTEN-deficient, chromosomal instability colorectal cancer is hypersensitive to STAT3 inhibition
Source: Int J Biol Sci. 2025 Oct 20;21(15):6633–48. doi: 10.7150/ijbs.111254 (PMC12640721; doi:10.7150/ijbs.111254)
Supplement: Supplementary file 1 — Supplementary figures and tables. [file ijbsv21p6633s1.pdf]

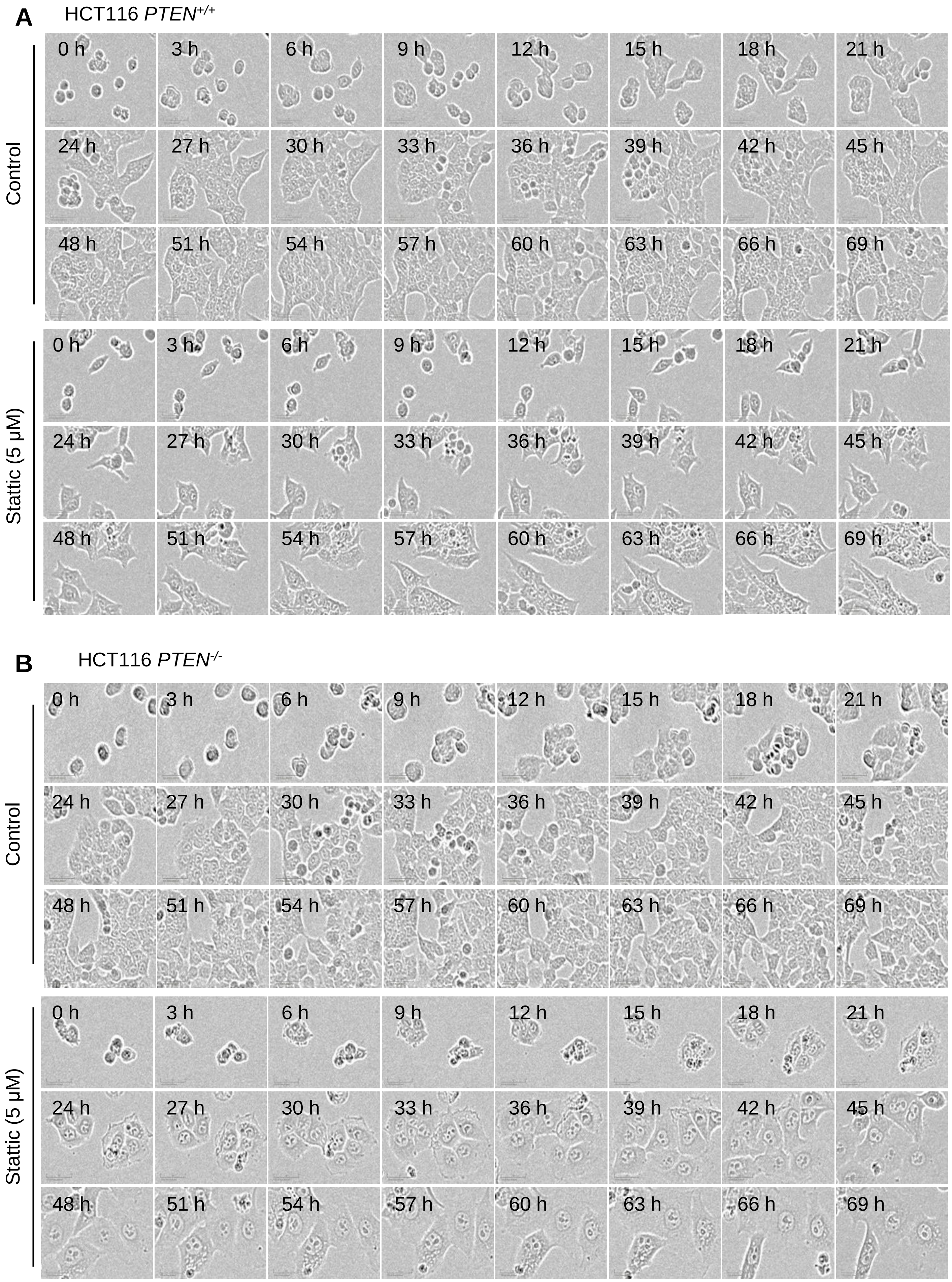

Supplementary Figure S1 Full panel images of HCT116 *PTEN*$^{+/+}$ and *PTEN*$^{-/-}$ cells with Stattic treatment. Images were taken at 3-hour intervals for a total of 69 hours.

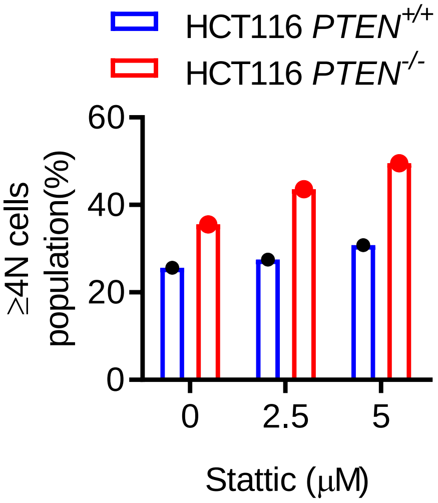

Supplementary Figure S2 Quantification of the cell population with DNA content bigger than 4N.

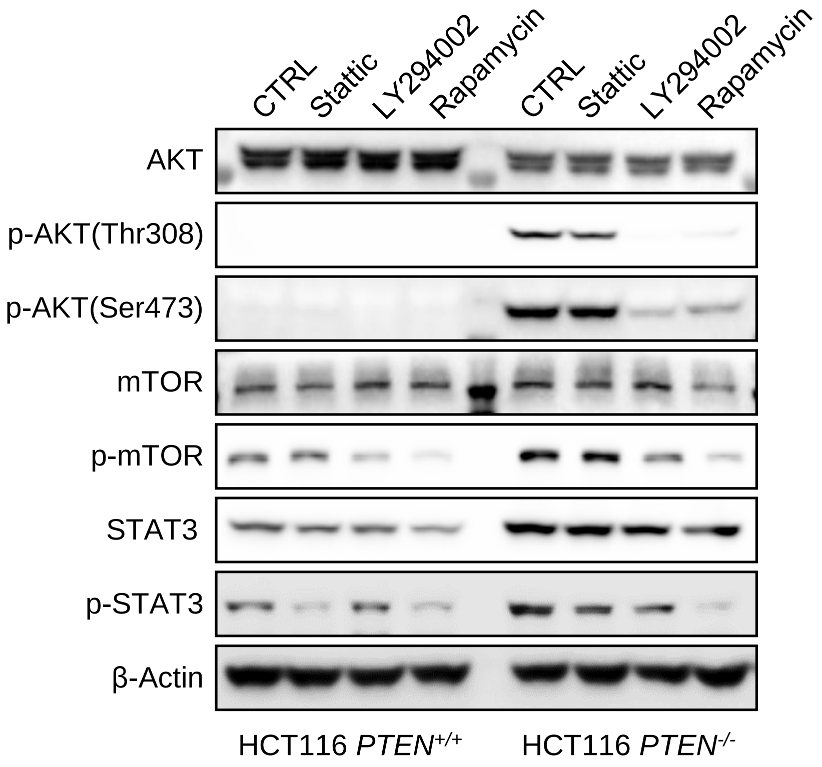

Supplementary Figure S3 Effects of PI3K/mTOR/AKT pathway on STAT3 signal in PTEN-deficient CRC cells. PTEN-isogenic HCT116 cells were treated with or without STAT3 inhibitor (Stattic), PI3K/mTOR inhibitor (LY294002), and mTOR inhibitor (Rapamycin) for 24 h and Western blots were conducted with total and phosphorylated antibodies for each protein.

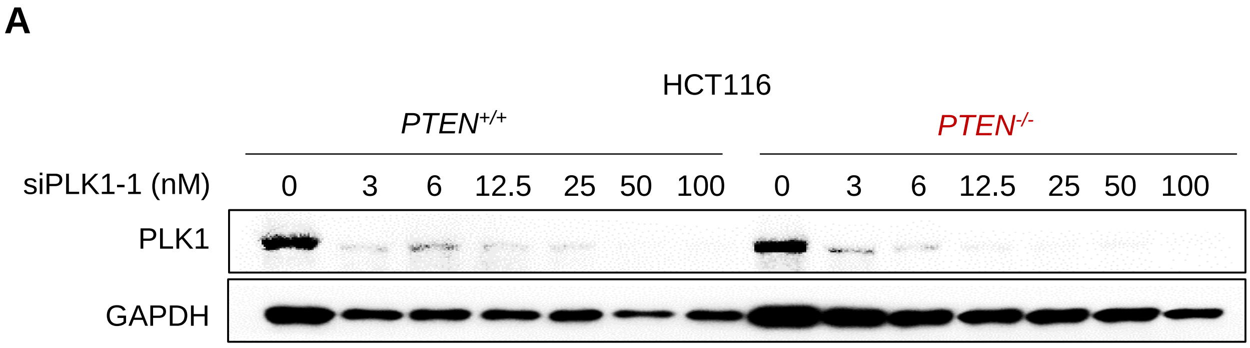

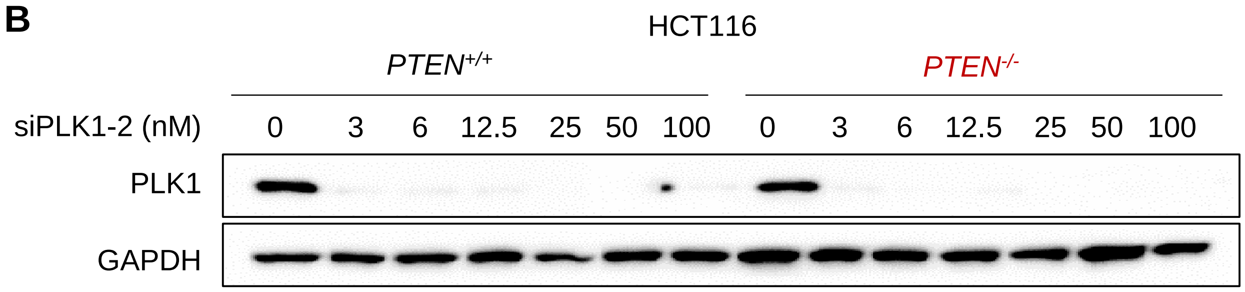

Supplementary Figure S4 Immunoblots showing the siPLK1-1 (A) and siPLK1-2 (B) knockdown efficiency in PTEN-isogenic HCT116 cells.

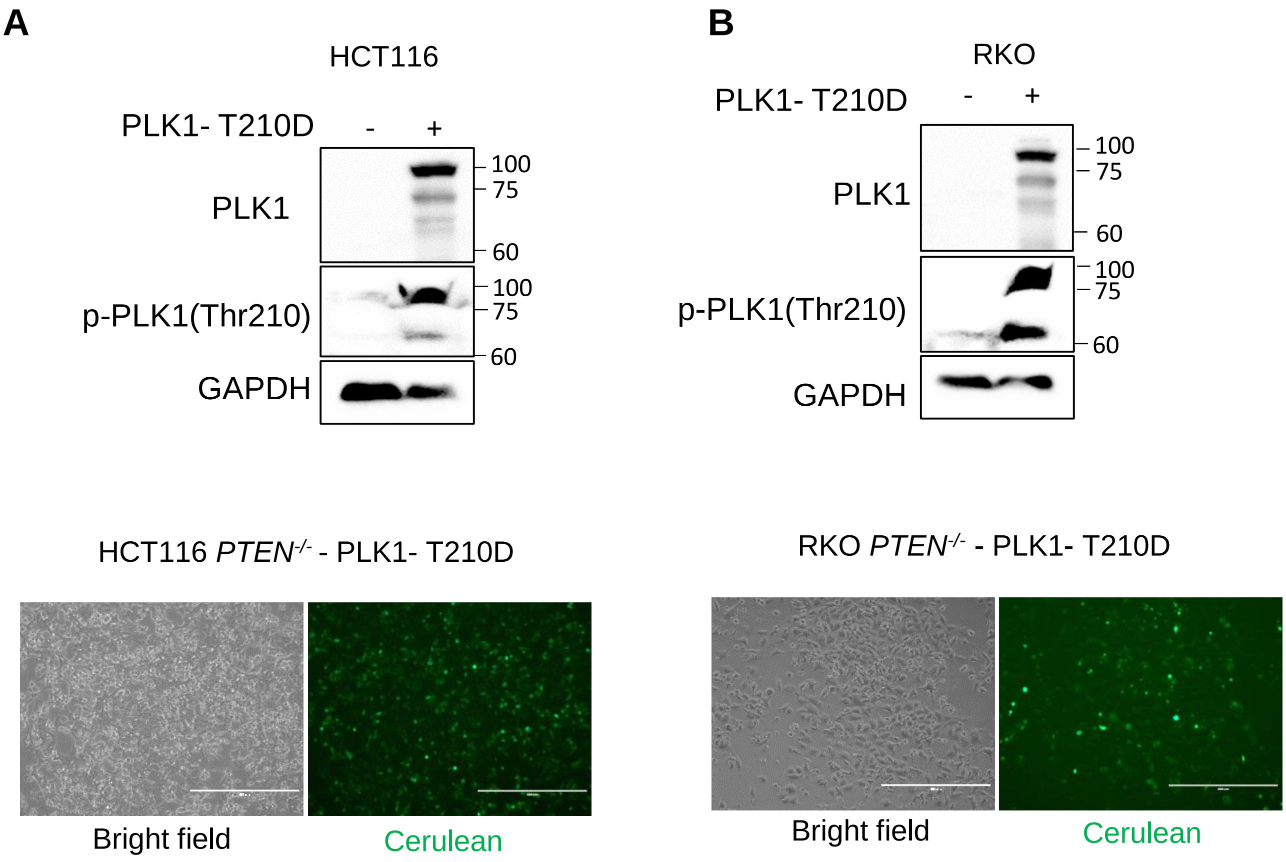

Supplementary Figure S5 Immunoblots and immunofluorescence images showing the Overexpression of $PLK1^{T210D}$, a constitutively active form of PLK1, in *PTEN*$^{-/-}$ HCT116 cells (A) and in *PTEN*$^{-/-}$ RKO cells (B).

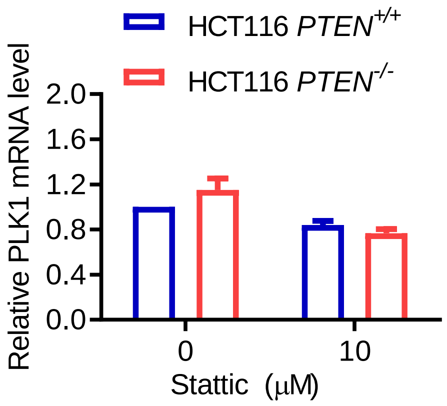

Supplementary Figure S6 PLK1 mRNA level in HCT116 *PTEN*$^{+/+}$ and *PTEN*$^{-/-}$ cells with 10 μM Stattic treatment

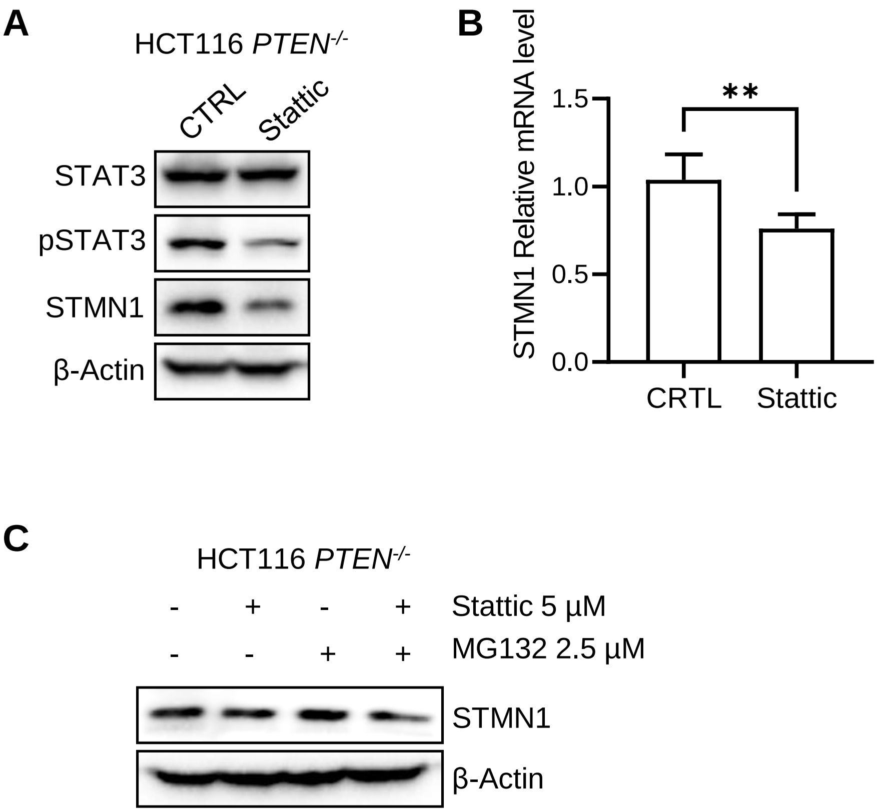

Supplementary Figure S7 The role of STAT3 in the regulation of STMN1. PTEN-deficient HCT116 cells were treated with or without Stattic for 24 h and the protein level (A) and mRNA level (B) of STMN1 were assessed. Data are presented as mean ± SD (n = 3 independent experiments), $^{**}P < 0.01$ between two indicated bars (unpaired t test). C.PTEN-deficient HCT116 cells were treated with Stattic or MG132 (proteasome inhibitor) alone or combination for 6 h and the protein level of STMN1 was assessed.

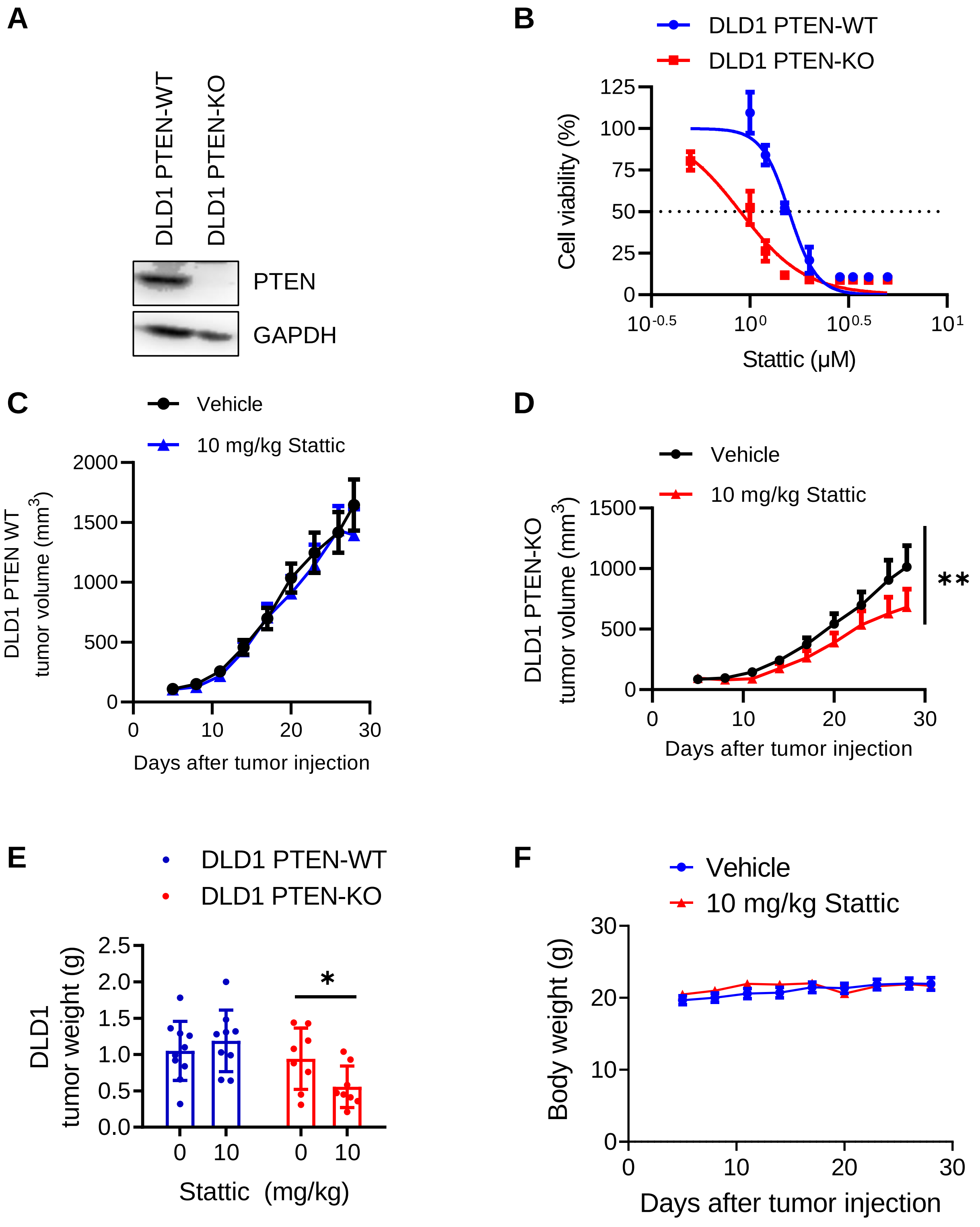

Supplementary Figure S8 Validation of the synthetic lethality in DLD1 PTEN-isogenic CRC cell pair. A. PTEN wildtype (WT) and knockout (KO) DLD1 cell lines generated by CRISPR/Cas9 was validated with Western blots of PTEN status. B. Differential drug sensitivity of Stattic on PTEN-isogenic DLD1 cell lines. Data are presented as mean ± SD (n = 3 independent experiments), C-E. In vivo effects of Stattic on PTEN-WT and KO DLD1 tumor xenografts. F. Effect of Stattic on mouse body weight. Data are presented as mean ± SD. *$P < 0.05$, **$P < 0.01$ between two indicated curves (two-way ANOVA test).

Supplementary Table 1. Antibodies used in this study.

No	Primary Antibodies	Suppliers	Catalog No.	Molecular weight (kDa)
1	PTEN (A2B1)	Santa Cruz Biotechnology	sc-7974	55
2	AKT (B1)	Santa Cruz Biotechnology	sc-5298	62
3	p-AKT (ser473)	Cell Signaling Technology	9271	60
4	p-AKT (thr308)	Cell Signaling Technology	9275	60
5	GAPDH	Santa Cruz Biotechnology	sc-365062	37
6	PARP-1 (H-250)	Santa Cruz	sc-7150	116
7	PLK1	Cell Signaling Technology	#4513	62
8	p-PLK1 (thr210)	Cell Signaling Technology	#5472	62
9	STAT3	Cell Signaling Technology	#14475	21
10	p-STAT3 (tyr705)	Cell Signaling Technology	#9145S	79
11	γ-tubulin	Santa cruz	sc-10732	50
12	STMN1	Cell Signaling Technology	#13655S	19
13	BUB1B	Thermo fisher	#MA1-16577	21
14	α-tubulin	Santa Cruz	sc-5286	46
15	MAD2L	Santa Cruz	17D10	34
16	Cyclin E	Cell Signaling Technology	sc-377100	53
17	mTOR	HUABIO	ET1608-5	289
18	p-mTOR	Cell Signaling Technology	5536	289
19	β-Actin	Santa Cruz	sc-47778	45

Supplementary Table 2. Sequences of siRNAs used in this study

No	Target gene	Suppliers	Sequence
1	STAT3	Integrated DNA Technologies	5’-AUCAUUGAGCCAAAUCUUAAAAAAA-3'
2	PLK1	Integrated DNA Technologies	5'-GCCUCUGGCAUUAGAAUUAUUUAAA-3’
3	STMN1	Integrated DNA Technologies	5'-ACAAAAUGGAAGCUAAUA-3’
4	BUB1B	Integrated DNA Technologies	5'-AUUCUAAAGGUCCCAGUGUACCUTT-3’
5	MAD2L	Integrated DNA Technologies	GGUUUUCCUGAAAUCAAGUCAUCTA-3’
6	AURKA	Integrated DNA Technologies	5'-CAAUUUCCUUGUCAGAAUCCAUUAC-3’

Supplementary Table 3. Sequences of qPCR primer pairs used in this study.

No	Target gene	Suppliers	Sequence
1	PLK1	BGI	Forward 5'-AATACACCAGCAAGCTAGATGC-3'<br>Reverse 5'-AATCAGTTCCGTTCCCCAGAG-3'
2	GAPDH	BGI	Forward 5'-GTGGACCTGACCTGCCGTCT-3'<br>Reverse 5'-GGAGGAGTGGGTGTCGCTGT-3'
